# Designing a Hybrid Energy-Efficient Harvesting System for Head- or Wrist-Worn Healthcare Wearable Devices

**DOI:** 10.3390/s24165219

**Published:** 2024-08-12

**Authors:** Zahra Tohidinejad, Saeed Danyali, Majid Valizadeh, Ralf Seepold, Nima TaheriNejad, Mostafa Haghi

**Affiliations:** 1 Department of Electrical Engineering, Ilam University, Ilam 69315-516, Iran; z.tohidinejad@ilam.ac.ir (Z.T.); m.valizadeh@ilam.ac.ir (M.V.); 2 Ubiquitous Computing Laboratory, HTWG Konstanz—University of Applied Sciences, 78462 Konstanz, Germany; ralf.seepold@htwg-konstanz.de; 3 Institute of Computer Engineering, Heidelberg University, 69120 Heidelberg, Germany; nima.taherinejad@ziti.uni-heidelberg.de

**Keywords:** energy harvesting, hybrid, medical wearable sensor nodes, health monitoring, power management

## Abstract

Battery power is crucial for wearable devices as it ensures continuous operation, which is critical for real-time health monitoring and emergency alerts. One solution for long-lasting monitoring is energy harvesting systems. Ensuring a consistent energy supply from variable sources for reliable device performance is a major challenge. Additionally, integrating energy harvesting components without compromising the wearability, comfort, and esthetic design of healthcare devices presents a significant bottleneck. Here, we show that with a meticulous design using small and highly efficient photovoltaic (PV) panels, compact thermoelectric (TEG) modules, and two ultra-low-power BQ25504 DC-DC boost converters, the battery life can increase from 9.31 h to over 18 h. The parallel connection of boost converters at two points of the output allows both energy sources to individually achieve maximum power point tracking (MPPT) during battery charging. We found that under specific conditions such as facing the sun for more than two hours, the device became self-powered. Our results demonstrate the long-term and stable performance of the sensor node with an efficiency of 96%. Given the high-power density of solar cells outdoors, a combination of PV and TEG energy can harvest energy quickly and sufficiently from sunlight and body heat. The small form factor of the harvesting system and the environmental conditions of particular occupations such as the oil and gas industry make it suitable for health monitoring wearables worn on the head, face, or wrist region, targeting outdoor workers.

## 1. Introduction

With the global spread of the coronavirus (COVID-19) disease, the importance of the remote monitoring of human health has been further highlighted. In this context, wearable devices have gained additional attention in healthcare [1,2]. These devices can seamlessly integrate into the daily lives of individuals, and through the continuous monitoring of vital signs, serve as an effective solution for early disease detection [3]. This early diagnosis can result in preventive measures and prompt therapeutic solutions, preventing the progression of the disease and reducing some of the costs associated with emergency and hospital care [4,5]. Additionally, the use of wearable devices in remote areas can enhance the efficiency of healthcare services, quality of life (QoL), and overall well-being [6]. With surveillance, assessment, and continuous data provision, these devices assist in the early detection of the users’ health issues, transforming into a promising approach for preventive healthcare [7].

Wearable technologies encompass electronic devices like smartwatches, wristbands, and augmented reality glasses, often including various physiological/non-physiological sensors, a data processing unit, and communication components. They can collect a diverse range of data including heart rate (HR), blood pressure (BP), respiratory rate, blood oxygen saturation (SpO_2_), body movement and physical activity, and more, by using sensors such as photoplethysmograms (PPGs) and accelerometers; these sensors serve as valuable tools in providing real-time health tracking data, especially for individuals dealing with chronic illnesses such as asthma, chronic obstructive pulmonary disease (COPD), diabetes, mobility impairments, and cardiovascular diseases, contribute to identifying movement-related disorders such as Parkinson’s disease, and are particularly useful for individuals dealing with Alzheimer’s disease, respectively [8,9]. In the realm of wearable technology, the proliferation of unobtrusive, non-intrusive, and non-invasive devices signifies a key advancement in user-centered design [10,11,12]. These features are particularly important in continuous health monitoring [13,14]. There is a hindrance in the practical use of wearable devices, stemming from issues related to battery recharging and battery life, among other aspects such as accuracy, reliability, and clinical use [6,15,16,17,18,19,20,21].

With the reduction in size and power consumption of electronic circuits, integrating data processing units, and communication components, real-time monitoring and analysis have been enhanced, which allow healthcare professionals to remotely monitor their patients’ progress and make informed decisions regarding their medical care [17].

Despite the above-mentioned advantages of using wearable devices and some of the most popular and contributing sensors with the applications, these electronic devices rely on batteries to power themselves [22]. Therefore, a primary concern in these scenarios revolves around the necessity of a continuous power source. This dilemma has led to the emergence of innovative solutions, particularly in the field of energy harvesting approaches and power management systems. Energy harvesting techniques involve capturing and utilizing environmental energy sources such as solar energy [23], thermal energy [24], vibrations [25], kinetic energy [26], and radio frequency (RF) [27] energy to supply power to wearable devices. Additionally, efficient power management systems optimize the use of available energy, ensure a longer battery life, reduce the need for frequent recharging, and store the harvested energy from the environment or the individual’s body in an energy storage unit, which is ultimately used to power the consumer load [28].

The power density of various energy sources is provided in Table 1.

Accordingly, solar energy in outdoor environments has the highest power. However, in many cases, it may not fully meet the needs due to the instability of these sources and their temporal-spatial unavailability. To address this issue, the use of hybrid energy harvesting systems has been introduced as an effective solution, capable of harvesting energy from multiple sources [29]. Hybrid energy harvesting systems have the advantage of providing a more reliable and stable power source by harnessing energy from various sources. By integrating various energy harvesters such as photovoltaic (PV) panels, thermoelectric generators (TEG), kinetic harvesters, RF harvesters, and more, these systems can ensure a continuous flow of energy, even when a single source is unavailable or not producing sufficient power. This not only increases the overall power density, but also enhances the system’s stability and efficiency [29].

Our contributions in this work are as follows:

- Designing a double-source hybrid PV/TEG energy harvesting system to achieve the maximum power point tracking (MPPT) for both input sources, during battery charging and loading, addressing 96% efficiency in energy conversion.
- Validating the harvesting system with two DC/DC boost converters, which operate with MPPT and can charge or supply a common battery/load simultaneously or individually, showcasing the capability to turn into a self-powered wearable device.
- Implementing a low-cost, compact form factor, and universal harvesting system compatible with a wide range of wearable healthcare devices in different mode of wearability such as wrist- or head-worn systems.

The rest of this paper is organized as follows. Section 2 reviews the related work. In Section 3, we describe the materials, methods, and study design. Section 4 presents the experimental results. This is followed by the discussion in Section 5, and we present out conclusions in Section 6.

## 2. Related Work

Several single-source and hybrid energy harvesting systems for healthcare monitoring applications have been introduced. Yaoguang et al. [30] proposed a wearable TEG that harvests heat from the human body. The structural architecture of the TEG device consisted of 12 TEG modules electrically linked in series and connected in parallel via copper strips. This wrist-worn TEG device captured body heat and generated more power while walking compared to stationary. A requirements analysis and performance evaluation of wearable sensors in medical applications was presented in [26], addressing the fundamental issue of piezoelectric kinetic energy harvesting devices. In [31], a kinetic energy harvesting device was used instead of an accelerometer to assess calorie consumption, as kinetic energy is generated when the user expends calories through bodily movements. A wearable sensor system for long-term health monitoring was described in [28], where the device measured the temperature, HR, SpO_2_, and human body acceleration in real-time. In [32], a rotating piezoelectric energy harvesting device was tested and developed, capable of generating a maximum power of 7 μW when worn on the arm during activities. Additionally, ref. [33] described the development of a flexible piezoelectric generator designed to harvest energy from the dynamic movement of the ear canal. In [34], a scalable triboelectric energy harvesting system for electronic textiles was proposed to extract energy from daily human movement. The suggested energy harvester is scalable, stretchable, and wearable. The system’s output power is enhanced by the capacitor capacity and the mechanical input frequency, providing guidance for practical applications. However, a drawback of this system is its ability to power wearable electronic devices only during human movement, with significant power losses due to high rectifier losses. In [35], a wearable sensor system in the form of glasses was proposed. This system employs algorithms to detect and identify chewing cycles using a piezoelectric pressure sensor placed on the temporalis muscle. The study suggests the potential for the further miniaturization of electronic devices to improve user comfort. Additionally, further research is needed to explore new methods of integrating sensors such as embedding them in the handles of glasses. In [36], a novel hybrid energy harvesting technology was presented to power wearable electronic devices. The study developed a flexible and wearable energy harvesting device that combined solar and RF energy. This work represents the first flexible and wearable hybrid system of solar and RF energy harvesting that was experimentally tested on the human body. Furthermore, to increase the reading range of active radio-frequency identification (RFID) tags and provide a compact multifunctional structure, a hybrid solar and RF energy harvesting system was introduced in [37]. This system includes components such as monocrystalline solar panels, a charging circuit, a rectifier, the EM4325 chip as the receiver antenna, and an RFID tag. The authors in [38] proposed a flexible TEG module that is appropriate for biomedical and wearable devices due to its high-power density on a small scale and flexibility due to its flexible form factor. In [39], various methods were examined to utilize the heat and mechanical energy of the human body for wearable energy harvesting. The focus was on harvesters such as TEG, PV, piezoelectric, electromagnetic, and electrostatic harvesters. This work included hybrid energy harvesters that hybrid the conversion of two or more energy sources to achieve the maximum power density.

Typically, in hybrid energy harvesting systems, Schottky diodes such as the 1N5817 are used to combine input sources, but they suffer from high power losses and voltage drops. This technique, known as “OR-ing”, can be applied either before or after the voltage conversion stage. Applying this technique before the voltage conversion stage allows for the use of a single voltage converter for both sources. However, it creates a parallel structure of energy sources and requires the sources to have the same internal impedance. As a result, the load is supplied by the energy source with the higher voltage until the other source surpasses it. Therefore, at any given time, only one energy source can be utilized [28,40,41].

To overcome this drawback, we present a hybrid energy harvesting system designed to power a wide range of wearable medical healthcare sensors including those worn on the head and wrist. The primary objective of this system is to design and implement a compact and efficient double-source energy harvester that simultaneously utilizes solar and body heat sources, thereby extending the battery and system lifespan. This energy harvester relies on compact PV panel and TEG modules. The collected energy is stored in a 3.7 V, 300 mAh lithium battery, facilitating system charging. Additionally, two ultra-low-power DC/DC boost converters are employed to efficiently manage and convert the generated power from the PV and TEG sources. These converters ensure that the harvested energy is effectively converted to the voltage and current levels required for the system load, optimizing the use of available energy and increasing the overall battery life. Furthermore, the use of diminutive PV panel and TEG modules contributes to a streamlined and lightweight design, making it well-suited for various wearable applications.

## 3. Materials and Methods

The design and implementation of the hardware harvesting system and health-related sensors and components include a PV panel, TEG module, lithium battery, and two BQ25504 (Texas Instruments, Dallas, TX, USA) ultra-low-power DC/DC boost converters, MAX30102 (Analog Devices, Norwood, MA, USA), and MPU6050 (TDK InvenSense, San Jose, CA, USA).

We considered two ultra-low-power DC/DC boost converters for the PV and TEG sources, with identical configurations for battery settings. The ultra-low-power DC/DC boost converter controls the output voltage of the PV panel and TEG module. The low-power IC BQ25504 is the basis for this converter and the proposed structure, accepting a maximum absolute input and output voltage range from −0.3 V to 5.5 V.

### 3.1. Health-Related Sensors

PPG is a non-invasive technique used to estimate vital signs such as HR, SPO_2_, and BP. The MAX30102 sensor provides a convenient and efficient solution for real-time monitoring. It comes in a compact form factor, has low power consumption initialization, a wide range of voltage support, and supporting inter-integrated circuit (I2C) protocol [42,43,44]. This sensor consumes 6 mA in measurement mode and 2.7 mA in its sleep mode. The MAX30102 chip has compact dimensions of 5.6 mm × 3.3 mm × 1.55 mm.

Another common example of sensors used in wearable technologies for health monitoring is the accelerometer. This can measure physical activity by detecting changes in acceleration. By using the gyroscope and accelerometer present in MPU6050, rotation along all three axes, static acceleration due to gravity, and dynamic acceleration due to motion can be measured. The current consumption is 4.8 mA when active.

### 3.2. Proposed Hybrid Energy Harvesting System, and Hardware Specification

**Figure 1:**
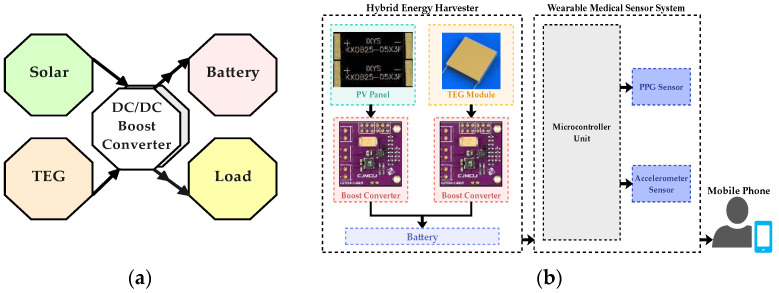
(**a**) The block diagram of the hybrid energy harvesting system; (**b**) proposed development of the hybrid energy harvesting system.

The proposed block diagrams of the hybrid energy harvesting system are shown in Figure 1a,b. The system includes a PV panel for solar energy harvesting, a TEG module for body thermal energy capture, and two DC/DC boost converters. These converters are independent at their input terminals to connect and boost one of the input DC voltages of the PV panel and TEG module. In the later stage, they become parallel at their two output terminals to connect a battery storage and microcontroller unit (NodeMCU Board (Espressif Systems, Shanghai, China)) as a load.

#### 3.2.1. Solar Panel

We chose solar cells, model KXOB25-05X3F (×10) from IXYS (Milpitas, CA, USA), each with dimensions of 23 mm × 8 mm × 1.8 mm and a maximum power of 30.7 mW under standard conditions: temperature of 25 °C and 1000 W/m^2^ irradiance. This PV panel consisted of a series and parallel connection of these ten cells with an area of 1840 mm^2^, every two cells were connected in series, and ultimately, five strings were connected in parallel. This model has a 25% power conversion efficiency. The electrical characteristics of this cell are given in Table 2. All values were measured at the standard condition: 1 sun (=100 mW/cm^2^), Air Mass 1.5, 25 °C.

#### 3.2.2. TEG Module

We used the IMC06-126-03 TEG (RMT Ltd, Moscow, Russia) module with dimensions of 16 mm × 16 mm and a thickness of 1.4 mm. The energy harvested from TEG depends on the performance parameters of the TEG module, the TEG module’s cross-sectional area, and the material’s Seebeck coefficient. In the temperature range of the human body, which is the hot side, the cross-section of the TEG modules must be increased to increase the generated power. Increasing the number of TEG modules effectively improves the cross-sectional area that contributes to power generation, allowing for larger power outputs. Connecting TEG modules in series is a useful solution. Four TEG modules chained in series formed the total area of 1024 mm^2^. The polarity of the TEG depends on the direction of the cold and hot sides. These TEGs were used to harvest energy from the body heat. The performance parameters are provided in Table 3 for the TEG cold side in dry air at 27 °C. During the experiments, measurements, and estimations in this work, we only considered the first column information as validation.

#### 3.2.3. DC/DC Converter and Power Management Unit

**Figure 2:**
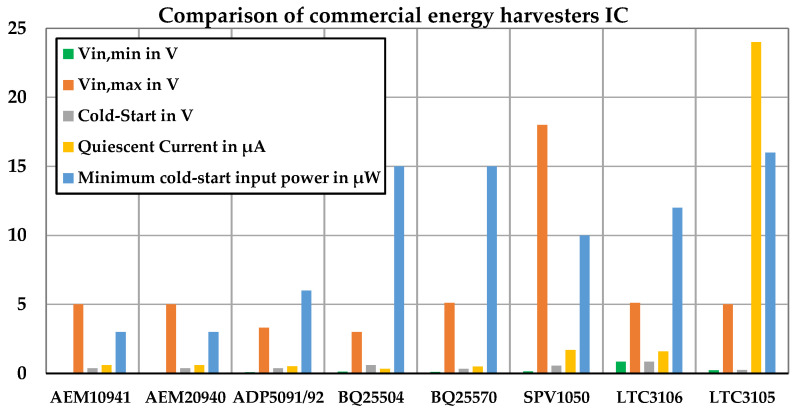
Comparison of several examples of commercial energy harvesting ICs used for PV/TEG energy sources.

The DC voltage produced by PV/TEG energy harvesters is typically low. DC/DC power converters such as boost converters were used in the power management circuit to increase the voltage to the required level. We identified and compared the influencing characteristics of several ICs from different companies such as Texas Instruments (TI), STMicroelectronics, Analog Devices, and E-peas on their relevance to the PV and TEG input sources (see Figure 2) [42,43,44,45,46,47,48,49].

A low quiescent current is crucial for maintaining efficiency in low-energy harvesting systems. According to Figure 2, BQ25504 and BQ25570 (Texas Instruments, Dallas, TX, USA) as well as AEM10941 and AEM20940 (E-peas, Mont-Saint-Guibert, Belgium) exhibited low quiescent current consumption, with BQ25504 being the most efficient. In comparison, ADP5091/92, LTC3105/06 (Analog Devices, Norwood, MA, USA), and SPV1050 (STMicroelectronics, Geneva, Switzerland) had a medium to high quiescent current. Taking into account other factors such as low prices and availability, we selected BQ25504.

#### 3.2.4. Energy Storage Unit

The harvested energy was stored in a rechargeable lithium battery with dimensions of 40 mm × 11 mm × 4 mm, capacity of 300 mAh, and a nominal voltage of 3.7 V, which reaches 4.2 V when fully charged.

### 3.3. Proposed Multi-Port Energy Harvesting Circuit

**Figure 3:**
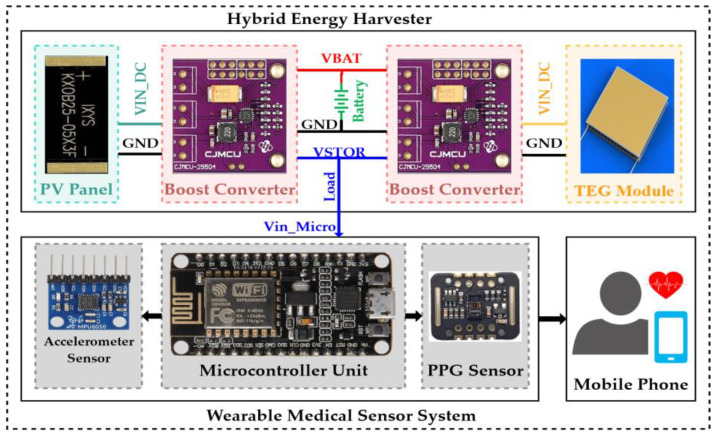
The proposed multi-port energy harvesting system.

The details of the proposed multi-port energy harvesting power circuit are shown in Figure 3.

**Figure 4:**
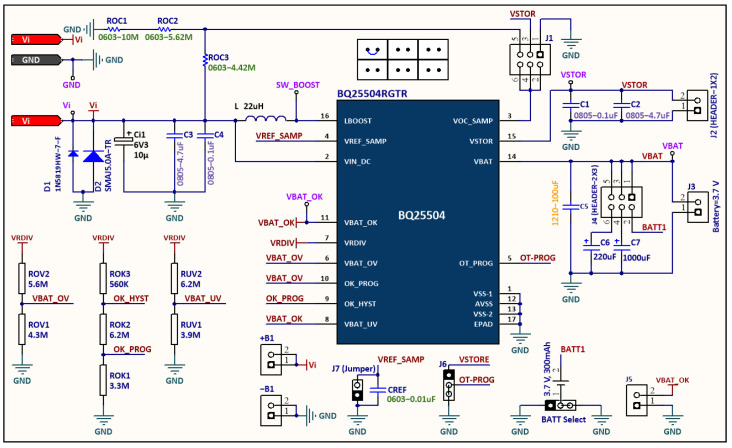
Circuit schematic of the BQ25504 ultra-low-power DC/DC boost converter.

Both the V_BAT_ and V_STOR_ pins of both converters were connected to each other (output parallel). As a result, a single battery terminal and a single load terminal were achieved by these common pins to integrate the system input battery and output load. The battery overvoltage (OV) and undervoltage (UV) configurations of both boost converters were designed to be the same (see Figure 4). Therefore, each power source operates with the MPPT and can charge/supply a common battery/load simultaneously or individually.

## 4. Experimental Results

The proposed sensor node included one MAX30102 sensor and one MPU6050 with two operating modes: active mode, in which MAX30102 was activated for 10 S at a sampling rate of 200 Hz, and sleep mode for 180 S. The MPU6050 module worked continuously with a sampling rate of 50 Hz. We tested and measured the battery lifetime of the sensor node under the condition that the energy harvesting system was disconnected. The total operation time of the node was (T). Therefore, in the active mode, the current consumption of the node was measured as: I_ON_ = 36 mA for a period of T_ON_ = 10 S. Furthermore, in sleep mode, we recorded the current consumption of I_Sleep_ = 32 mA during T_Sleep_ = 180 S. Thus, the average current consumption was I_ave_ = 32.21 mA.

With the node’s operating voltage of 3.3 V, the power and energy were 106.29 mW and 382.6 Joules, respectively. As a result, with the battery capacity of 300 mAh, the total battery lifetime (T_BAT_) was calculated as 300 mAh/32.21 mA = 9.31 h.

To calculate the energy produced by the PV panel (E_PV_), we considered three environmental conditions in which a subject/user wore the sensor node and worked comfortably.

- Sunny day facing the sun;
- Sunny day back to the sun;
- Shady or cloudy conditions.

We performed the experiments for 10, 60, and 120 min (see Table 4).

Assume that a worker in the oil, gas, or petrochemical industry is engaged in outdoor activities for 4 h in direct sunlight: 2 h with their back to the sun, and 2 h in shaded conditions. The average power of the PV panel in direct sunlight is 235.5 mW; when this person is engaged in activities with their back to the sun, it is 140 mW; and in shaded conditions, the power is 10.25 mW. Therefore, the E_PV_ can be calculated using Equation (1), which equals 4472.88 joules.

(1) $$E_{PV}=P_{PV}\times t\;E_{PV}=\left[\left(2.94\;V\times 80.1\;mA\times 4\;h\right)+\left(2.8\;V\times 50\;mA\times 2\;h\right)\;+\left(2.18\;V\times 4.7\;mA\times 2\;h\right)\right]\times 3600\;S=4472.88\;Joules$$

The battery has a capacity of 300 mAh, and the maximum voltage is 4.2 V. Therefore, using Equation (2), the battery has a stored energy of 1260 joules, where C_BAT_ is the battery capacity and V_BAT_ is the battery voltage.

(2) $$E_{BAT}=C_{BAT}\times V_{BAT}\;E_{BAT}=300\;mAh\times 4.2\;V=1260\;mWh$$

The estimated charging time (T_CH_) of the battery by the PV energy harvester is measured as:

(3) $$T_{CH}=E_{BAT}/E_{PV}\;T_{CH}=1260/4472.88=0.28\;day=6.72\;h$$

In the same manner, the output energy of the TEG module is measured as follows:

(4) $$E_{TEG}=P_{TEG}\times t_{TEG}$$

In a scenario where the output power of the TEG module (P_TEG_) is considered as 82.2 mW for a period of 8 h, the generated TEG energy at a temperature difference (∆T) of 8 °C (∆T = T_hot_ − T_cold_ = 35 °C − 27 °C = 8 °C) can be calculated as E_TEG_ = 2367 joules. Table 5 shows the details of the TEG module test conditions.

### 4.1. The Results of the Hybrid Energy Harvesting System

**Figure 5:**
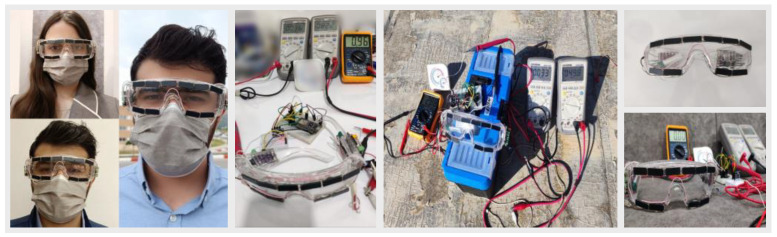
Hardware implementation of the prototype energy harvesting system on glasses as a wearable device, which are worn on the human body.

The conversion efficiency of the PV panel harvester was calculated at the maximum measured input power of 1840 mW. This amount was 1000 W/m^2^ of sunlight intensity, and according to the measured PV panel area of 0.01840 m^2^, the maximum peak power output at the standard conditions was 307 mW, and the conversion efficiency was 16.68%. The system energy harvesting prototype is shown in Figure 5, when it is worn on the human body.

We also evaluated the PV and TEG energy harvesting system under various resistive loads. Each step is explained below.

#### 4.1.1. First Experimental Stage: Wearable Sensor Node

**Figure 6:**
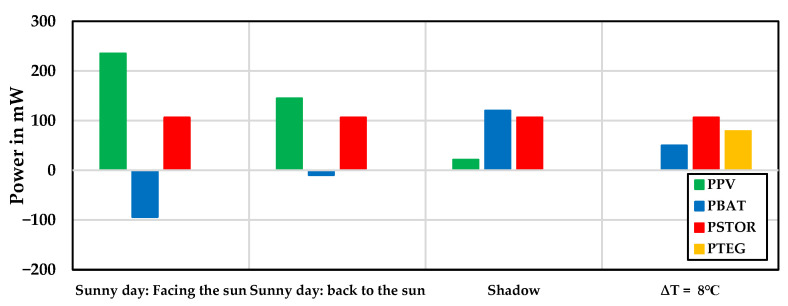
Exchange of PV/TEG power, battery, and wearable sensor node power in different weather conditions.

Figure 6 shows the maximum measured power of the PV energy harvester at different hours on 27 and 28 August 2023, respectively (for further details, see Table 4). The measurements were taken under various conditions: a sunny day facing the sun, a sunny day with the back to the sun, and a shadow day where P_TEG_ = 0. According to this comparison, negative battery power indicates that the battery is charging, while positive battery power indicates that the battery is discharging, meaning that power consumption is being supplied from the battery.

We estimated the losses of the BQ25504 sensor according to the input and output power and then calculated the efficiency as follows:

(5) $$P_{IN}=P_{loss}+P_{OUT}$$

(6) $$\eta =\frac{P_{OUT}}{P_{IN}}=\left\{\begin{array}{c} if\;P_{BAT}>0\;\to \;\left\{\begin{array}{c} {P_{OUT}=P}_{STOR}\\ P_{IN}=P_{PV}+P_{TEG}+{|P}_{BAT}| \end{array}\right.\\ if\;P_{BAT}<0\;\to \left\{\begin{array}{c} {P_{OUT}=P}_{STOR}+{|P}_{BAT}|\\ P_{IN}=P_{PV}+P_{TEG} \end{array}\right. \end{array}\right.$$

The maximum conversion efficiency on a sunny day facing the sun (ƞ) was 85%, where ƞ is the ratio of output power to input power. The PV energy harvester charges the battery and provides a current for the sensor node. On a sunny day with the back to the sun, the PV panel alone supports the total power required for the sensor node, effectively turning it into a self-powered system without the battery (see Table 4). The maximum conversion efficiency in this scenario was 80%.

According to Figure 6, the PV panel could not fully meet the sensor node’s power consumption in shadow conditions. Therefore, the battery acted as a backup, supplying the additional power required by the sensor node.

Figure 6 also shows the TEG module test conditions at a temperature difference of 8 °C, with P_PV_ = 0. In this scenario, the battery supplemented the system load alongside the TEG module. The maximum conversion efficiency of the TEG module (ƞ) was 82%.

#### 4.1.2. Second Experimental Stage: PV Energy Harvesting System under the Various Resistive Loads

**Figure 7:**
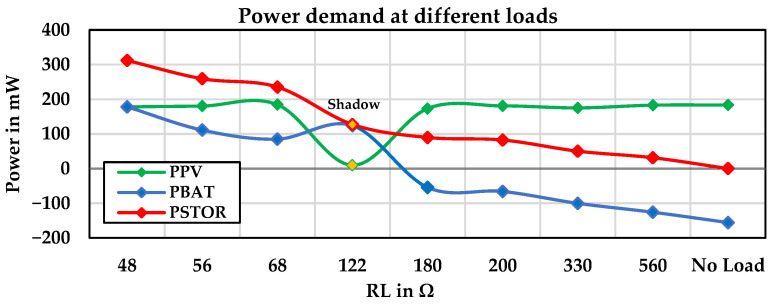
The power demand of the PV energy harvesting system under the various resistive loads.

Figure 7 illustrates the values of the PV input power, battery power, and output power under various resistive loads on a sunny day. It also shows the person entering into the shade for a few minutes under specific conditions and then returning to the sunlight.

**Figure 8:**
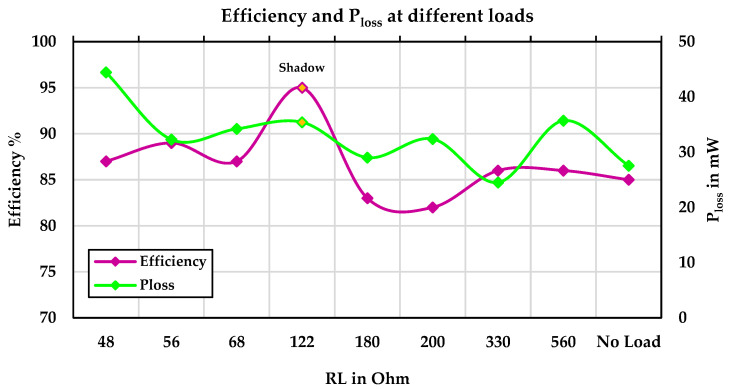
Efficiency and power losses of the PV energy harvesting system under various resistive loads.

According to the diagram, in the no-load state, the energy harvested from the PV panel is stored in the battery. As the resistive load values are varied from 31 mW to 312 mW, the battery is charged at five points, achieving an efficiency of 83%. At other points, due to the high load demand, the battery discharges, resulting in an efficiency of 87%, as the system’s power consumption is supplemented by the battery. Figure 8 shows the efficiency and power losses of the PV energy harvesting system under various resistive loads on sunny days and specifically in shadow conditions.

#### 4.1.3. Third Experimental Stage: TEG Energy Harvesting System under the Various Resistive Loads

**Figure 9:**
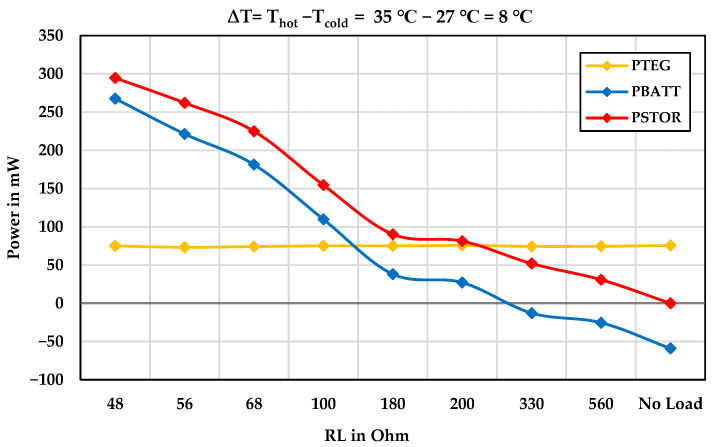
The power demand of the TEG energy harvesting system under the various resistive loads.

**Figure 10:**
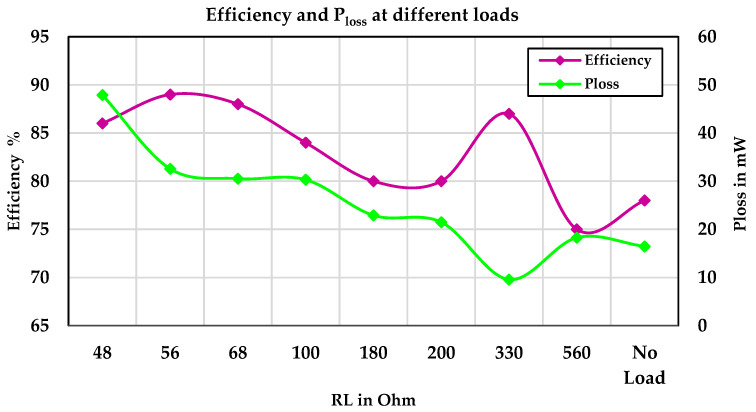
Efficiency and power losses of the TEG energy harvesting system under various resistive loads.

Figure 9 illustrates the TEG input power, battery power, and output power under various resistive loads at a temperature difference of 8 °C. At three points, the battery is also being charged in addition to the load being supplied by the TEG modules. Figure 10 shows the efficiency and power losses of the TEG energy harvesting system under various resistive loads at a temperature difference of 8 °C.

#### 4.1.4. Fourth Experimental Stage: Hybrid Energy Harvesting

**Figure 11:**
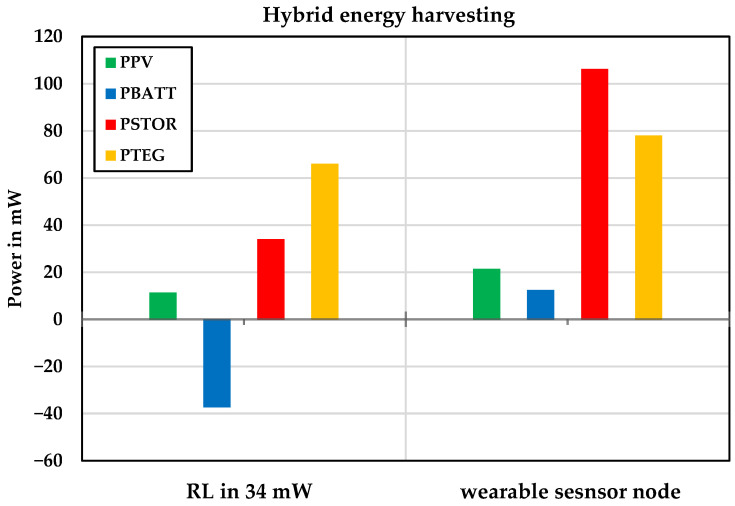
The contribution of hybrid energy harvesting resources in supplying the output load in shadow conditions.

Figure 11 shows the contribution of hybrid energy harvesting sources under shade conditions and a temperature difference of 8 °C to supply a wearable sensor node and a 34 mW load. The battery was charged in addition to supplying the load, achieving a system efficiency of 92%. Furthermore, the system efficiency with the wearable sensor node was 95%.

**Figure 12:**
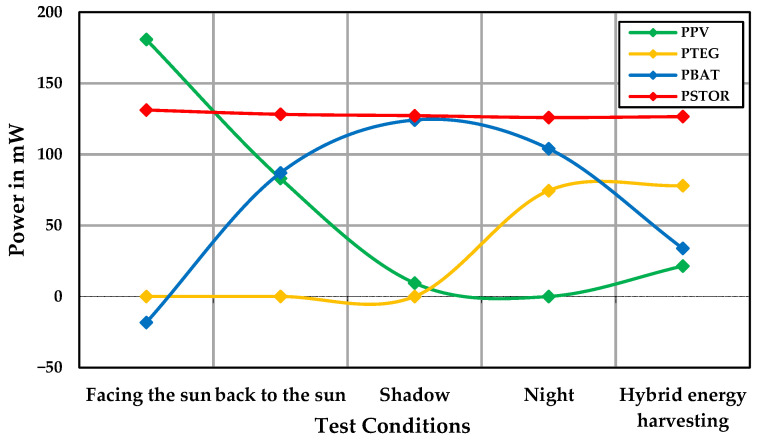
Power contribution of the hybrid energy harvesting system for wearable sensor node.

Figure 12 compares the hybrid energy harvesting system under partly cloudy conditions and a temperature difference of 8 °C. According to this diagram, MPPT was achieved in the hybrid structure of both energy harvesting sources.

**Figure 13:**
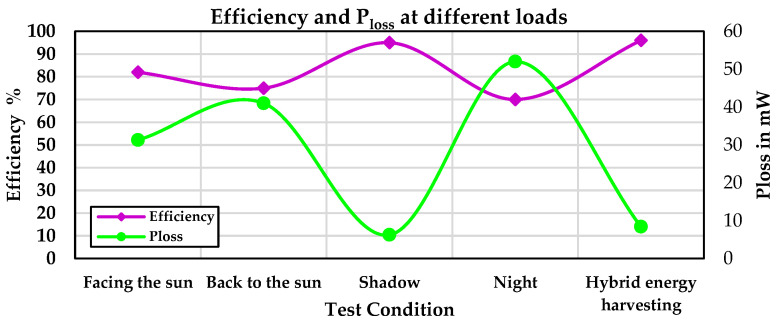
Energy harvesting system efficiency and P_loss_.

Figure 13 shows the efficiency and P_loss_ of the system and TEG module at a temperature difference of 8 °C, and the hybrid energy harvesting system under shadow conditions with a temperature difference of 8 °C. In the shadow condition, the battery provided most of the output power, resulting in high system efficiency. In the hybrid energy harvesting system, the system efficiency was 96%, with the battery providing only a small portion of the consumed power.

## 5. Discussion and Comparison

We designed and developed a hybrid double-source energy harvesting system to support wearable devices for health monitoring in the head, face, and wrist regions, focusing on long-term continuous measurement. We primarily targeted individuals engaged in outdoor activities such as workers in the oil and gas industry. Given the high-power density of solar cells outdoors, a hybrid of PV and TEG energy was utilized to harvest energy quickly and sufficiently from sunlight and body heat. Since body heat is an inherent part of the human body, TEG can provide a useful energy source for the wearable device when sunlight is not available or when a person is indoors, considering the temperature difference of the available energy source. This improves the temporal-spatial stability and reliability of the harvesting system.

The system harvests input powers from PV and TEG sources simultaneously, utilizing two BQ25504 low-power DC/DC boost converters to supply the load and charge the battery. By eliminating Schottky diodes with high power losses and voltage drops, and only harvesting energy from the source with the higher voltage at any given moment, this structure can enhance the system’s performance. Additionally, the PV panel and TEG modules support small form factor and high efficiency, which are essential in providing the ease of use, user experience, and unobtrusiveness of wearable devices.

Table 6 shows the comparison of the proposed hybrid energy harvesting system with the related previous works [28,30,50,51]. In any wearable device, the total dimension and form factor play pivotal roles in the user experience, useability, and practicability that lead to unobtrusiveness. Thus, careful circuit design and component selection are vital due to the limited space. Considering these points, we tried to reduce the overall area of the PV and TEG energy harvesting system, which was much lower compared to other works. Consequently, this can facilitate further wearing wearable devices by users and drive them toward the wear-and-forget. Although our careful design and component selection resulted in shrinking the size of the PV panel and TEG module, this reduction in dimensions did not decrease the output power of the PV panel and TEG module. The harvesting sources could achieve an appropriate output power from the solar sources and body heat, which can support powering the sensors and wearable devices and store excess harvested energy in batteries—in the ideal condition. The power consumption of the systems depends on various factors including the types of sensors. The other influencing factor in assessing the total energy consumption is the MCU/embedded system. For example, low-power MCUs such as nRF52840 (Nordic Semiconductor, Trondheim, Norway), MSP430FR5969 (Texas Instruments, Dallas, TX, USA), ADuCM302/ADuCM305 (Analog Devices, Wilmington, MA, USA), and STM32L4 (STMicroelectronics, Geneva, Switzerland) can extended the battery life. However, due to our focus on the energy harvesting system itself and its suitability for integration in wearable devices, along with considerations of the cost and availability, we used the NodeMCU microcontroller. Although this microcontroller has higher power consumption compared to other low-power options, the total energy consumption was not prioritized. This choice does not reflect the actual efficiency of the system, as it could simply be impacted by the microcontroller’s higher power demands.

When harvesting energy from multiple sources, some kind of OR-ing structure is needed. This can be carried out before or after the voltage conversion step [40,41]. While using the first structure has the advantage of using a single voltage converter for both sources, it limits the power sources to having the same internal impedance, and only one source can be used at a time. Therefore, using two BQ25504 ultra-low power boost converters with the same configuration, we made it possible to achieve simultaneous energy harvesting from input sources and separate the MPPT for each source, which did not require an additional diode in the output part, and reduced the power loss.

Although the continuous measurement of physiological and non-physiological parameters is of concern to all groups of occupations and health, however, some of the targeting group could take priority due to several reasons such as safety and harsh environmental conditions that expose them to more frequent risks. Those in the oil and gas industry are such workers, and the continuous monitoring would provide them with several advantages. For instance, it is known for its hazardous working conditions including exposure to toxic chemicals, high-pressure equipment, extreme temperatures, and physically demanding tasks. Wearable health monitors can help identify potential health risks and provide real-time alerts in case of emergencies, enhancing worker safety. Furthermore, fatigue is a major concern in the industry—not only in oil and gas, but long working hours can also lead to decreased alertness and cognitive function. Wearable devices can track sleep patterns and activity levels to help employers and workers manage fatigue effectively, reducing the risk of accidents. Additionally, wearable devices can continuously monitor vital signs like HR, body temperature, and respiratory rate. This enables the early detection of health problems such as heat stress or cardiac issues, allowing for timely intervention and the prevention of more serious health events. Monitoring the health of workers in the oil and gas industry through wearables is crucial for enhancing safety, preventing accidents, complying with regulations, and improving overall worker well-being and productivity.

Therefore, considering the condition in which our proposed system was worn by outdoor workers exposed to sunlight for two hours (in the actual situation of workers in the oil and gas industry, this period is longer), the energy consumption of the wearable sensor node was turned into a self-powered system. In this situation, surplus energy was also stored in the battery for times when energy harvesting sources were not available. However, one of our main limitations was in evaluating the system in real conditions in the workplace, particularly in the oil and gas industry. Additionally, convincing individuals to wear these glasses during work poses a challenge. The application of this system can be considered not only for workers in the oil and gas industry, but also for other individuals such as mountaineers and those interested in health monitoring.

Wearable electronics, despite their negligible individual power consumption, significantly impact the global energy usage due to their sheer numbers. In 2022, 1.1 billion wearable healthcare devices, consuming an average of 656 mW each (including hub consumption), accounted for 727 MW of power and 1942 tons of CO_2_ emissions, necessitating 11.34 million conifer trees to offset. Given their exponential growth, it is crucial to design more energy-efficient wearables. Implementing power harvesting techniques could mitigate their environmental footprint and reduce the burden on the energy sector [52].

In future work, it is possible to expand energy harvesting input sources and provide a multi-input hybrid structure (e.g., body motion energy or RF ambient energy). Furthermore, integrating health monitoring sensors such as those for checking the blood glucose levels of individuals with diabetes, skin temperature for detecting fever, and more. Additionally, the use of flexible components, which bring a lot of comfort to wearable devices, can be explored.

## 6. Conclusions

One of the main challenges in the continuous and unobtrusive measurement of health-related parameters by wearable biomedical sensors is the battery’s capacity and form factor. Energy harvesting techniques (single/multisource) are widely used to extend the lifetime of wearable nodes. We designed and implemented an efficient hybrid PV/TEG energy harvesting system in a compact, low-cost form factor that is compatible with wearables worn on the face, head, and wrist. This system prolongs measurement and supports the continuous monitoring of physiological parameters.

Our meticulous design and component selection resulted in a smaller PV panel and TEG module without reducing their output power. The PV panel conversion efficiency of our proposed system was 16.68%, significantly higher than the previously reported 4.79%. At the core of the system, we utilized two BQ25504 DC/DC boost converters, both active and employed simultaneously. Unlike previous studies using the OR-ing structure, our design allowed each power source to operate with MPPT, charging/supplying a common battery/load simultaneously or individually.

Experimental results demonstrated the feasibility of the overall design, doubling the sensor system’s battery lifetime to 18 h. The efficiency of the hybrid energy harvesting system was 96%, enabling the system to become self-powered under direct sunlight for two hours. Our results indicate that the system could be further extended for use in outdoor occupations.

## Figures and Tables

**Table 1:** Power densities of different energy sources [6].

Energy Sources	Power Density
Ambient light	100 mW/cm^2^ (direct sun) 100 μW/cm^2^ (indoor illumination)
Thermoelectric	60 μW/cm^2^
Radio frequency	1 μW/cm^2^ (ambient) 15 μW (external)
Human	1000 μW/cm^2^ (biochemical) 4 μW/cm^3^ (biomechanical—microgenerator) 200 μW/cm^3^ (biomechanical—piezoelectric)

**Table 2:** KXOB25-05X3F electrical characteristics.

Symbol	Cell Parameter	Typical Ratings
V_OC_	Open circuit voltage	2.07 V
I_SC_	Short circuit current	19.5 mA
V_mpp_	Voltage at MPP	1.67 V
I_mpp_	Current at MPP	18.4 mA
P_mpp_	Maximum peak power	30.7 mW
H	Solar cell efficiency	25%

**Table 3:** 1MC06-126-03TEG performance data.

Symbol	Parameter	Values at Hot Side Temperature		
		35 °C	55 °C	85 °C
T_cold_	Cold side temperature, (°C)	27	27	27
Opt_η_	Optimum efficiency, (%)	0.40	1.36	2.71
P_OPT_	Optimum power, (mW)	20	233	964
V_OPT_	Optimum voltage, (V)	0.244	0.868	1.825
V_OC_	Open circuit voltage, (V)	0.43	1.51	3.18
I_SC_	Short circuit current, (A)	0.19	0.63	1.24

**Table 4:** PV panel testing conditions on a sunny day and measured energy.

Test Conditions	Sunny Day: Facing the Sun			Sunny Day: Back to the Sun			Shadow		
	10 min	1 h	2 h	10 min	1 h	2 h	10 min	1 h	2 h
V_PV_ in V	2.92	2.91	2.94	2.8	2.7	2.8	2.09	2.38	2.18
I_PV_ in mA	71.2	63.4	80.1	51.7	48.5	50	5.3	9	4.7
P_PV_ in mW	207.9	184.5	235.5	144.76	131	140	11.07	21.42	10.246
V_BATT_ in V	3.75	3.96	3.96	3.83	3.81	4.04	3.93	3.87	3.81
I_avrage_ in mA ^1^	32.21	33.21	33.21	33.26	34.15	32.21	28.21	32.21	28.21

^1^ [(I_STOR-ON_ × T_active-Sensor_) + (I_STOR-OFF_ × T_sleep-Sensor_)]/T.

**Table 5:** Details of the TEG module testing conditions in temperature difference ∆T= T_hot_ − T_cold_ = 35 °C − 27 °C = 8 °C.

Test Conditions	Indoor	
	10 min	1 h
V_TEG_ in V	0.96	0.96
I_TEG_ in mA	82.2	82.2
P_TEG_ in mW	78.912	78.912
V_BATT_ in V	3.93	3.92
I_avrage_ in mA ^1^	32.21	33.21
Skin temperature	35	35
Environment temperature	27	27

^1^ [(I_STOR-ON_ × T_active-Sensor_) + (I_STOR-OFF_ × T_sleep-Sensor_)]/T.

**Table 6:** Comparison of the current work with some previous studies.

Ref.	Energy Source	Sensors Deployed	Energy Storage	Area of Harvester (mm^2^)	Power of Harvester (mW)	Mode of Device Wearability	Energy Management IC	MCU Unit	Circuit Techniques for Hybrid
This work	PV, TEG	PPG, Accelerometer	Battery, 300 mAh	Panel = 1840, TEG = 1024	Panel = 307, TEG = 78.2 at (∆T = 8 °C)	Glasses, Wrist-worn	Two BQ25504 boost converters	NodeMCU ESP8266	Energy harvesting from both sources, without diode
[28]	PV, TEG	Temperature, Pulse oximeter, Accelerometer	Supercapacitor, 50 F	Panel = 4320, TEG = 1600	Panel = 207, TEG = 50 at (∆T = 20 °C)	Wrist-worn	LTC3105 boost converter	ATmega-328p	Power OR-ing.
[30]	TEG	Powering a LED	N/A	TEG = 559	TEG = 0.023 at (∆T = 10 °C)	Wrist-worn	LTC3108 boost converter	N/A	__
[50]	PV	N/A	Battery, CR2025-supercapacitor, 4 F	40,000	820	N/A	BQ25570 buck-boost converter	Atmel ATMEGA328P-AU	__
[51]	PV, TEG	Nano-power accelerometer, Temperature, Analog microphone	Battery, 40 mAh	Panel = 3892, TEG = 560	Panel = 4.42, TEG = 2.62 at (∆T = 16 °C)	Bracelet	BQ25570 buck-boost converter and LTC3108 boost converter	MSP430FR5969	Energy harvesting from both sources, with diode

## Data Availability

No new data were created or analyzed in this study. Data sharing is not applicable to this article.
